# Diffuse X-ray scattering from polished silicon: application of the distorted wave Born approximation

**DOI:** 10.1107/S1600577522000534

**Published:** 2022-02-08

**Authors:** Albert Macrander, Lahsen Assoufid, Suresh Narayanan, Ruben Khachatryan

**Affiliations:** a X-ray Science Division, Advanced Photon Source, Argonne National Laboratory, Argonne, IL 60439, USA

**Keywords:** diffuse X-ray scattering, surface roughness, power spectra, computational modeling

## Abstract

Measured diffuse X-ray scattering data for a ‘smooth’ as well as for a ‘rough’ silicon sample were fit to theoretical expressions within the distorted wave Born approximation (DWBA). Data for the power spectral density (PSD) for both samples were also obtained by means of atomic force microscopy and optical interferometry. The Fourier transforms of trial correlation functions were fit to the PSD data and then applied to the DWBA formalism. The net correlation functions needed to fit the PSD data for each sample comprised the sum of two terms with different cutoff lengths and different self-affine fractal exponents. At zero distance these correlation functions added up to yield net values of σ^2^ = (2)^2^ and (71)^2^ Å^2^ for the smooth and rough samples, respectively. X-ray scattering data were obtained at beamline 1-BM of the Advanced Photon Source. Data and fits at values of *q*_*z*_ = 0.05 and 0.10 Å^−1^ for the smooth sample are reported. Good fits for the smooth sample were obtained at both *q*_*z*_ values simultaneously, that is, identical fitting parameters were applied at both values of *q*_*z*_. The smooth sample also exhibited weak Yoneda wings and a clear distinction between the strong specular scattering and the weak diffuse scattering. Data for the rough sample were qualitatively different and exhibited very weak scattering at the specular condition in contrast to extremely large Yoneda wings. Fits for the rough sample are reported for *q*_*z*_ = 0.04, 0.05, and 0.06 Å^−1^. Although the large Yoneda wings could be fit quite well in both position and amplitude, scattering near the specular condition could not be equally well fit by applying the same fitting parameters at all values of *q*_*z*_. Albeit imperfect, best-fitting results at the specular condition were obtained by invoking only diffuse scattering, that is, without including a separate theoretical expression for specular scattering.

## Abstract 2

Measurements of diffuse X-ray scattering from polished silicon were fit. The fits employed autocorrelation functions obtained from direct metrology of the surface roughness.

## 1. Introduction

**Figure 1:**
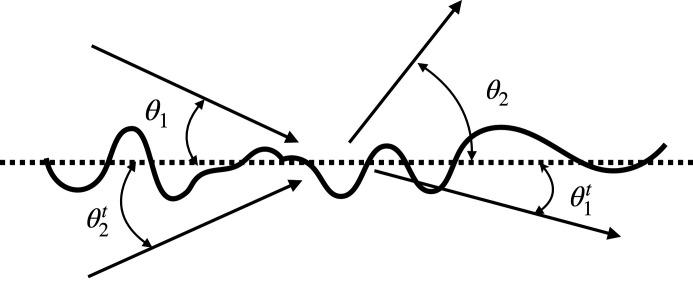
Schematic of a rough surface. The average surface is shown as a dashed line. The incident wave is incident at angle θ_1_; its corresponding transmitted wave is at angle 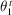 . The scattered wave is at angle θ_2_ and corresponds to a time-reversed state in DWBA. It has a corresponding inside wave at angle 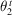 . The *Z*-axis is normal to the average surface.

Diffuse scattering of hard X-rays is a powerful technique to characterize surface roughness not only in amplitude but also in spatial frequency (Sinha *et al.*, 1988 ▸; Daillant & Gibaud, 1999 ▸). We report measurements for the surface of silicon as a study of polishing for X-ray optical applications at synchrotron beamlines, but the method can also be applied to soft matter (Tolan, 1999 ▸) as well as to mesoscopically patterned semiconductor surfaces (Schmidbauer, 2004 ▸). Theoretical expressions applicable to diffuse X-ray scattering from surface roughness were developed in a key paper by Sinha *et al.* (1988 ▸). Not only expressions for the Born approximation (BA) but also for the distorted wave Born approximation (DWBA) were developed (Schiff, 1968 ▸) in this paper. In the present work, the DWBA expression was applied to fit diffuse scattering data taken by rocking a sample at a specific value of *q*_*z*_, the vertical component of the scattering vector given by 

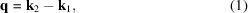

where the wavevectors **k**_1_ and **k**_2_ are the incident and scattered wavevectors (for waves above the surface), respectively. The expression requires as input a correlation function given by 

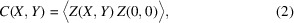

where *Z* is the height of the surface above an average surface. A rough surface as well as an average surface are schematically depicted in Fig. 1 ▸. In the treatment of the diffuse scattering, the surface is assumed to be isotropic and dependent only on the separation distance, *R* = [(*X* ^2^ + *Y* ^2^)]^1/2^, between two points in the average plane. Here, *X* and *Y* are Cartesian distances. The brackets in the expression for *C*(*X*, *Y*) denote a configurational average over a Gaussian distribution. The Wiener–Khinchin theorem (Ogilvy, 1991 ▸) relates the Fourier transform of *C*(*X*, *Y*) to the power spectral density of the surface.

A novel aspect of the present X-ray diffuse scattering study is that the power spectral density (PSD) was separately measured. Prior to fitting to the X-ray data, *C*(*X*, *Y*) = *C*(*R*) was obtained by fitting trial expressions for *C*(*R*) to the PSD data.

Sample preparation is discussed in Section 2. Surface profile measurements and power spectral densities are discussed in Section 3. Expressions for *C*(*R*) and the resulting fits to the PSD data are reported in Section 4. Following the treatment of Sinha *et al.* (1988 ▸) we give expressions for the BA in Section 5, and for the DWBA in Section 6. The diffuse scattering data were obtained at beamline 1-BM of the Advanced Photon Source. The experimental arrangement employed at 1-BM are detailed in Section 7. Finally, fits to the diffuse scattering data for both the smooth and rough sample are reported in Section 8.

## 2. Sample preparation

The starting material for both samples were cut from the same ingot of single silicon crystal grown by the float zone technique, and were 6 mm thick and 100 mm in diameter. The large surfaces of the slabs were oriented parallel to (111) crystallographic planes within 0.3 arcsec accuracy by X-ray diffraction method. Oriented slabs then were fine-ground using 9 µm-sized aluminium oxide abrasive and were etched in a solution of hydrofluoric and nitric acids. The etching process removed about 100 µm of damaged crystal. After the etching, slabs were first polished mechanically by using 1 µm-sized diamond slurry to achieve 3.5–4.0 nm r.m.s. nominal surface roughness. Subsequently, they were polished with chemical–mechanical polishing (CMP) (Babu *et al.*, 2001 ▸). CMP was performed with an IC-1000 type pad following the bowl feed technique (Dietz & Bennett, 1966 ▸). 50 nm-sized colloidal silica was used as a polishing compound.

## 3. Surface profile measurements and PSD

The PSD function represents the spatial spectrum of the surface roughness measured in inverse length. The PSD has been discussed in many excellent references (Bennet & Mattson, 1999 ▸; Stover, 1995 ▸; Elson & Bennett, 1995 ▸; Church, 1979 ▸; Duparré *et al.*, 2002 ▸). Practically, it is derived from calculating the square of the Fourier transform (FT) of the surface profile. In an ideal case, the PSD curve includes the entire surface spatial frequency range from zero to infinity, and all of the important surface parameters such as r.m.s. roughness, r.m.s. slope, and the correlation length can be obtained from moment integrals of the PSD (Ogilvy, 1991 ▸). However, for measured data there are bandpass considerations. For a two-dimensional continuous data set, the PSD is defined as (Church & Berry, 1983 ▸) 

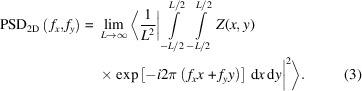

In order to connect to conventional expressions for PSD functions, in equation (3) we reverted back to a more general notation that does not assume a dependence on only the separation distances, *X* and *Y*. *Z*(*x*, *y*) is the surface as a function of surface coordinates *x* and *y*, and *f*_*x*_ and *f*_*y*_ are the spatial frequency in the *x* and *y* directions, respectively. We note that here we have followed the convention prevalent in the optical community of using direct spatial frequencies that differ from the reciprocal-space variables used below which are prevalent in the X-ray scattering community by a factor of 2π. That is, direct spatial frequencies must be multiplied by 2π to yield reciprocal-space quantities. *L* is the length of the measured area, and the brackets denote the ensemble average. For a linear surface profile *Z*(*x*) the PSD is defined as 

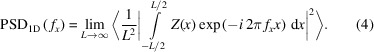

The subscripts 2D and 1D on the left-hand side of equations (3) and (4) above denote two-dimensional and one-dimensional PSDs, respectively. Surface profiles measured by any one technique are known over a limited number of discrete points. Therefore, the knowledge of the PSD curve over a wide range of spatial frequencies requires the use of different instruments and techniques to yield data with overlapped PSD ranges. The various PSD curves were concatenated into a single data set covering the entire measured frequency range and fit with a ninth-order polynomial function to yield an overall measured PSD profile.

### 3.1. Surface profile measurements

Measurements of surface profiles were obtained using both an atomic force microscopy (AFM) and an optical interference microscope.

**Figure 2:**
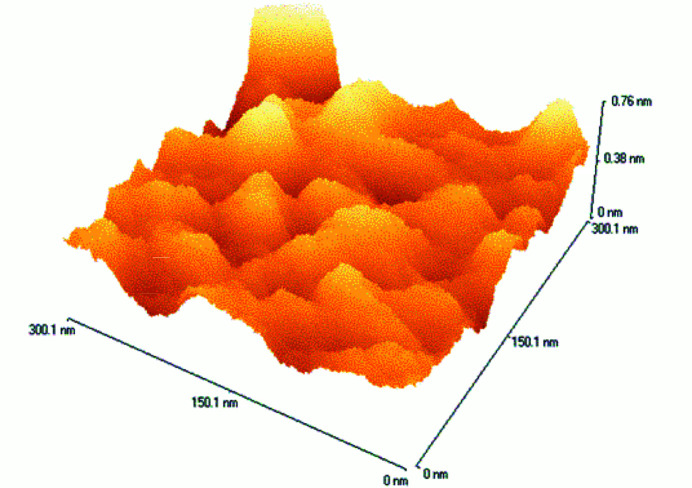
An image of the smooth sample obtained with an atomic force microscope.

The AFM instrument that we used was a TopoMetrix Explorer AFM (Topometrix Corp., Santa Barbara, CA, USA). Two different scanners were used: a tripod-type scanner, which has a maximum scan area of 130 µm × 130 µm, and a tube-type scanner, which has a maximum scan area 2 µm × 2 µm. To ensure a maximum overlap of PSD profiles, we chose to probe areas that are 300 nm × 300 nm, 2 µm × 2 µm, and 6 µm × 6 µm. The latter was measured using the tripod scanner while the first two were measured with the tube scanner. The surfaces were scanned in a raster mode with the sharp tip (∼10 nm diameter) of a microfabricated Si cantilever, model 1650-00, commercially available from Topo­metrix, that was mounted at the tip of the PZT scanner. The system was operated in noncontact mode, and the cantilever resonance frequency was approximately 230 kHz. Surface topography data consisted of 500 points by 500 points, which led to sampling intervals of 0.6 µm, 4 µm, and 12 µm for the chosen scan sizes, respectively. Example AFM images for both the smooth and rough samples are shown in Figs. 2 ▸ and 3 ▸, respectively. The image was corrected for piston, tilt, and curvature.

The interference microscope is a Wyko (Wyko Corp., Tucson, AZ, USA) model Topo-2D interferometer (Wyant, 1987 ▸). The instrument operates at a 630.3 nm wavelength (obtained through spectral filtering of a 50 W tungsten-filament light source). The measurements are based on the well known phase shifting interferometry technique (Creath, 1988 ▸). The length scale sensed depends on the objective lens used to perform the measurement. We used 1.5×, 5×, and 40× objectives lenses, which cover profile lengths of approximately 8 mm, 2.6 mm, and 333 µm, respectively. The 1.5× objective lens (data reported presently only for the smooth sample) is a Michelson interferometer, and the 5× and 40× objective lenses are based on a Mirau interferometer configuration. The data are collected on a very low noise linear detector array containing 1024 pixels. This results in sampling intervals of 8.7 µm, 2.5 µm, and 0.3 µm for the 1.5×, 5×, and 40× lenses, respectively. The maximum profile lengths of the objective lenses set the lower limit of measurable spatial frequency, and the upper limit is set by the Nyquist sampling resolution on the surface of the sample, which is equal to twice the value of the sampling interval. The data are digitized to 12 bits, so that each measurement is good to 1 part in 4095, and the instrument provides data with a height resolution of 0.01 nm and a repeatability of better than 0.003 nm under a controlled environment. However, the resolution is finally limited by the surface roughness of the reference mirror. The procedure for surface profile measurements is as follows. The sample surface was measured at ten randomly chosen locations. Each measurement was a result of averaging four measurements on the same spot. The main source of systematic errors is the roughness of the reference mirror, which typically has a surface roughness of several Å r.m.s. To account for these systematic errors, the profile of the reference surface was characterized using a highly polished SiC calibration mirror, which has a surface roughness of about 0.05 nm r.m.s. Then the obtained reference surface was stored in the computer memory and was subsequently subtracted from each profile. The averaging process minimizes random errors. The resulting one-dimensional height profiles were used to calculate the corresponding PSD profiles. The calculated PSD data are averaged to obtain the final PSD. Measurements were averaged over multiple randomly chosen locations for a more representative PSD of the sample surface.

### 3.2. Calculation of the PSD

The PSD data presented in this work were obtained in a digitized form with a finite number of data points. Topo-2D data are a 1D profile, *Z*(*x*), with 1024 points, and AFM data are in a 2D surface profile form, *Z*(*x*, *y*), with 500 × 500 data points. Therefore, the power spectra were calculated using discrete forms of equations (3) and (4) (Bennet & Mattson, 1999 ▸; Church & Takacs, 1993 ▸). For a 1D profile containing *N* data points, the PSD is given by

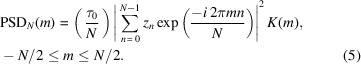

Here PSD_*N*_ is the discretized PSD calculated from a profile of *N* data points, τ_0_ is the spacing between data points, and *K*(*m*) is a bookkeeping factor that is equal to 1 except that *K* (±*N*/2) = 1/2 at the end of the power spectrum (Bennet & Mattson, 1999 ▸). Generally, a window function is added in the summation to smooth the ends of the finite-length data set to eliminate spurious effects in the spectral estimate that would otherwise appear (Bennet & Mattson, 1999 ▸; Church & Takacs, 1993 ▸).

Light scattering, in general, including X-ray scattering, can be related to the two-dimensional PSD of the surface (Church *et al.*, 1979 ▸), and we converted 1D PSD obtained from the Wyko-Topo-2D measurements into the corresponding 2D PSD. For isotropic surfaces, a procedure for conversion has been proposed by Duparré *et al.* (2002 ▸) and Church & Takacs (1991 ▸). Here we choose the procedure described by Duparré *et al.* (2002 ▸). First a 1D PSD is calculated for each measured profile on the surface using the *TOPO* routine developed by D. Windt (Windt, 2007 ▸). Then the obtained 1D PSDs are averaged for each sample. The averaging helps reduce measurement error and yields a better representation of the surface statistical parameters. The resulting averaged 1D PSDs are subsequently converted into the corresponding 2D isotropic PSD using the following expression, which is less sensitive to noise (Duparré *et al.*, 2002 ▸),

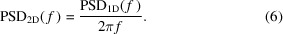




For the AFM measurements, the measured 2D surface height data were converted to radially averaged PSD profiles for each sample, using a computer routine developed by Windt (2007 ▸) that is similar to the procedure used by Stover (1995 ▸). We obtained radially averaged values for the PSD by converting frequencies to 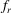 = 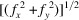 for *f*_*r*_ values ranging from the minimum spatial frequency to the maximum spatial frequency along *x* or *y* (whichever is smallest). The *i*th value of the radially averaged PSD, at a specific *f*_*r*_(*i*), is equal to the average of all PSD values contained in the annulus defined by 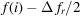 < 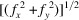 < 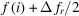 where Δ*f*_*r*_ is a spatial frequency increment. The result is an averaged 2D isotropic PSD. For each sample, the various radially averaged PSD data were concatenated into a single data set covering a wide range of radial spatial frequencies *f*_*r*_ and shown after the frequency scale was converted to a frequency in reciprocal space, that is, by applying 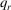 = 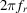 . Care has been taken to exclude data that exhibited a known drooping artifact arising from out-of-range spatial frequencies for a given type of measurement.

Due to a frequency-dependent transfer function, the Wyko-Topo-2D data attenuated the upper half of the optical band pass (Church & Takacs, 1991 ▸). This frequency-dependent effect is generally amenable to analysis and correction (Church & Takacs, 1988 ▸). At the lower-frequency end, distortions in the spectrum can result from data windowing and detrending. In particular, detrending effectively introduces a cut-off into the spectral domain that can be mis­interpreted as a finite correlation length in fractal surfaces (Church & Takacs, 1988 ▸). In this work, no restoration or correction was applied to the Wyko-Topo-2D data. Instead, we rely on the sufficient overlap between neighboring spectra of different objectives and instruments, and on the fact, as suggested by Church & Takacs (1988 ▸), that the lower end of one spectrum should become the higher end of the neighboring spectrum.

**Figure 4:**
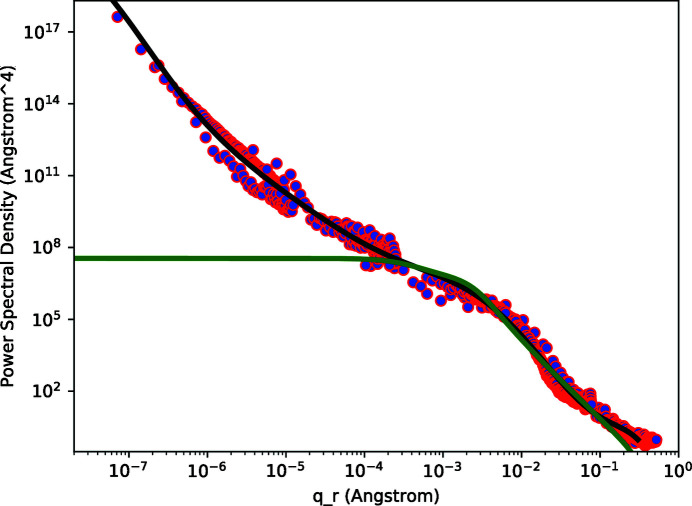
Power spectral density of the smooth sample. The data points were obtained from optical interferometry and from atomic force microscopy. The solid black curve is a polynomial fit to the concatenated data set. The solid green curve results from equation (8) applied to the correlation function shown in Fig. 6 ▸.

The concatenated data for both the AFM and Wyko instruments are shown in Figs. 4 ▸ and 5 ▸ for the smooth and rough samples, respectively. Polynomial functions fit to the PSD data are also shown in Figs. 4 ▸ and 5 ▸.

## 4. Correlation functions

The autocorrelation functions (referred to presently as correlation functions), denoted *C*(*R*), were obtained by trial and error by fitting Fourier transforms of trial functions to the polynomial fits to the PSD data. Sinha *et al.* (1988 ▸) treat a correlation function of the following form, 

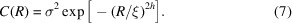

The parameters in this expression, σ, ξ, and *h*, are referred to as the roughness, cut-off length, and Hurst parameter, respectively. The Hurst parameter can be related to Hurst analyses of self-affine fractal surfaces (Russ, 1994 ▸). Values of *h* are positive definite and less than unity, with lower values corresponding to more ‘jagged’ surfaces, whereas values approaching unity correspond to ‘smoother’ surfaces that can be more accurately described as having ‘hills and valleys’ (Sinha *et al.*, 1988 ▸). Values of ξ can be compared with either the length of the X-ray beam footprint, the projection onto the surface of spatial coherence length of the X-rays, or an inherent property of the surface roughness. The question as to which of these is more appropriate was discussed by Sinha *et al.* (1988 ▸). This question is addressed for the present cases below.

We applied the following formula to convert trial expressions of *C*(*R*) to trial PSD functions for fitting purposes, 

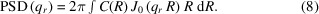

However, this expression applied to equation (7) results in only a single ‘knee’ in the PSD function. We find that the PSD data and the X-ray diffuse scattering data are better fit with a sum of such terms, and we applied the following expression, 

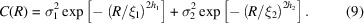

The final correlation functions which we used for subsequent fitting to diffuse scattering data are shown in Fig. 6 ▸ for both the smooth and rough samples. The values for the parameters in the correlation functions are listed in Table 1 ▸. The resultant PSDs via equation (8) are also shown in Figs. 4 ▸ and 5 ▸.

## 5. Scattering in the BA

In this section we reprise relevant X-ray scattering expressions within the BA as derived by Sinha *et al.* (1988 ▸). The index of refraction as most often denoted is given by 

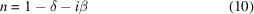

where the imaginary term accounts for photoelectric absorption. The critical angle for total external reflection, θ_c_, satisfies the following equation, 

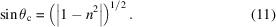

The critical angle determines the rocking angle at which Yoneda wings occur in diffuse scattering data (Sinha *et al.*, 1988 ▸). This dependence allows a determination of the index of refraction, and we have obtained values of the index of refraction in our fitting procedure as presented below.

As given by Sinha *et al.* (1988 ▸) the cross-section for diffuse scattering is given by 

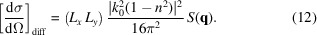

Here, *k*_0_ = 2π/λ, where λ = 1.23985 Å and *L*_*x*_ and *L*_*y*_ are the dimensions of the X-ray beam footprint on the sample along the beam direction and transverse to it, respectively. *S*(**q**) is given by 

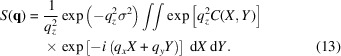

Here, *q*_*z*_, *q*_*x*_, *q*_*y*_ are the Cartesian projections of the scattering vector, **q**.

For an isotropic surface, *S*(**q**) can be written as 

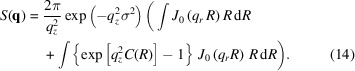

Here the net scattering has been split into a first term for specular scattering and a second term for diffuse scattering. We report below fitting to the specular term in equation (14) near *q*_*r*_ = 0, but instead of the diffuse term in equation (14), which applies in the BA, we have applied an expression applicable within the DWBA for the diffuse scattering.

## 6. Scattering in the DWBA

The diffuse scattering cross-section within the DWBA is given by 

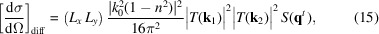

where, unlike in the case of the pure BA, two extra factors equal to Fresnel transmission factors must be included (Sinha *et al.*, 1988 ▸). These are given by 

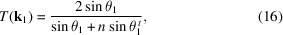




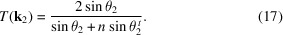

Here the angles are as defined in Fig. 1 ▸. Furthermore, 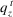 is the *z*-component of 

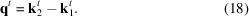

Here 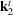 and 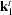 are the subsurface wavevectors incident at 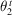 and 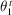 as depicted in Fig. 1 ▸. We note that, owing to X-ray absorption, **q** ^*t*^ is imaginary.

For *S*(**q** ^*t*^) we have applied the following expression which is rewritten from an expression given by Sinha *et al.* (1988 ▸), 

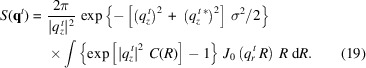

We note that because boundary conditions require that in-plane components of the scattering vectors must be preserved, we have that 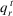 equals *q*_*r*_. The significance of this equivalence is that the PSD results obtained from measurements made to the outside surface, as detailed previously, and the resulting values of *C*(*R*) can be applied to equation (19).

## 7. Experimental arrangement

Diffuse scattering measurements were carried out at bending-magnet beamline 1-BM at the Advanced Photon Source. X-ray scattering measurements were carried out using a Huber four-circle diffractometer in the vertical scattering geometry. The optical layout of the beamline was as follows (Lang *et al.*, 1999 ▸). The first optical element was a Pd-coated mirror with a critical energy of 24 keV set to collimate the beam in the vertical direction. The radiation was monochromated using a double-crystal monochromator that also focused the beam sagittally with a dynamically bent second crystal. A horizontal fan of 2.6 mrad from the bending magnet was collected and focused onto the experimental station to a spot size of ∼0.7 mm full width at half-maximum (FWHM). A second Pd-coated mirror focused the beam vertically to a spot size of ∼0.3 mm (FWHM) at the sample position. A doubly focused incident beam at an energy of 10 keV (wavelength λ = 1.2398 Å) was used for the experiment. The detector was a scintillation counter directly preceded by a slit at 1 m distance from a sample. This detector slit was set to be wide open horizontally and set to a vertical aperture of 0.2 mm. Diffuse scattering data were taken by rocking the sample at a fixed *q*_*z*_ setting.

## 8. Diffuse scattering results

The following equation should be applied to compare calculated results to measured data (Sinha *et al.*, 1988 ▸),

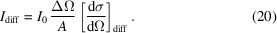

Here *I*_0_ is the intensity of the incident beam and *A* is the area of its cross-section, and ΔΩ is the solid angle intercepted by the detector slit. However, the above equation should more properly be stated in terms of a beam footprint, and *A* should be written as *L*_*y*_ times 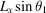 . However, since the sample size is limited, a walk-off condition may be reached as θ_1_ is decreased. In the present experiments, the incident vertical beam size was ∼0.3 mm and the critical angle was 3.22 mrad. This leads to a value for *L*_*x*_ equal to 93 mm at the critical angle. This is roughly the same as the sample sizes. As a result, a 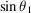 factor stemming from equation (20) was incorporated for θ_1_ only down to θ_c_. For lower incident angles, the value of θ_c_ was applied.

In order to apply the above scattering formalism we need to apply a single net value for the roughness, and we applied the value resulting from the following equation, 

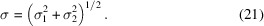

We concentrate here on relatively low values of *q*_*z*_, specifically, *q*_*z*_ ≤ 0.10 Å^−1^. X-rays penetrate more deeply at higher values of *q*_*z*_ which would raise additional complications, for example, having to account for roughnesses at the interface between a possible surface oxide layer and the underlying silicon. By concentrating on low *q*_*z*_, we can more confidently limit any theoretical treatment to the top surface and ignore any buried interface.

### 8.1. Smooth sample

The X-ray scattering data for the smooth sample are shown in Figs. 7 ▸ and 8 ▸ for *q*_*z*_ values of 0.05 and 0.10 Å^−1^, respectively. Also shown are simulations for specular scattering and diffuse scattering as well as the sum of these. The specular scattering was simulated based on the BA according to equation (14), and the diffuse scattering was simulated based on the DWBA according to equations (15)–(19). Both the measured data and the simulated specular peak were scaled to the Fresnel reflectivity within the BA at the exact specular condition (*q*_*r*_ = 0). The fitting parameters that we applied are listed in Table 1 ▸. The DWBA simulated results were scaled to optimize the agreement between the total simulated scattering (specular plus diffuse) and the measured data.

The overall *q*_*z*_ dependence was investigated by making a so-called longitudinal diffuse scan. This was done by rocking the sample with the *z*-axis rotated by 0.05° around the axis transverse to the diffraction plane. At low *q*_*z*_ such a scan picks up scattering in the specular peak, but the scan transitions to measuring pure diffuse scattering at higher *q*_*z*_. As shown in Fig. 9 ▸, this behavior was indeed found. Good agreement with the measured data and the summed specular and diffuse scattering calculations can be seen in this figure. Here we scaled the two calculated terms to optimize the overall agreement. The conclusion is that in addition to the good fits shown in Figs. 7 ▸ and 8 ▸ the overall *q*_*z*_ dependence resulting from our procedure of using the BA term in equation (14) for specular scattering and equations (15)–(19) for diffuse scattering in the DWBA approximation can be reproduced.

For the specular scattering the upper limits for the integral in equation (14) were 46 µm for the case of *q*_*z*_ = 0.05 Å^−1^ and 23 µm for the case of *q*_*z*_ = 0.10 Å^−1^. The FWHM of the specular peaks directly leads to these values. We note that both these values are much larger than the cut-off lengths discussed above. On the other hand, they are much smaller than the footprint of the beam on the surfaces of the samples which were several centimetres in length. They correspond instead, in both cases, to a distance of 0.23 µm transverse to the beam, which we interpret as the transverse coherence length of the incident X-rays. We conclude, furthermore, that the cut-off lengths in equation (9) are not set by the beam properties but are instead properties of the surface roughness. The slit before the detector determines the resolution in *q*_*r*_ presently, and the specular simulations were convolved with a Gaussian slit function to account for the limited resolution.

As discussed above, for extremely low incidence angles, the beam can be expected to have ‘walked-off’ the sample in the vicinity of the Yoneda peak on the grazing incidence side (*q*_*r*_ < 0). We found that best fits resulted under the assumption that this walk-off condition was reached at an incidence angle equal to the critical angle. Another point to note is that equation (20) implies an overall asymmetry with a somewhat lower intensity at grazing exit than at grazing incidence. This asymmetry was noted by Sinha *et al.* (1988 ▸) and arises from a sine relationship between the beam dimension and the footprint length. As can be judged from Figs. 7 ▸ and 8 ▸, the asymmetry in the data is reproduced.

We note that the rocking positions of the Yoneda peaks are very well reproduced in the fits. In order to emphasize this point, we again show in Fig. 10 ▸ the data and full fits, but for both *q*_*z*_ cases in the same figure. The index of refraction that yielded the position of these peaks is given in Table 2 ▸. This index is similar to that of a surface of pure silicon. This is a somewhat surprising finding since the samples had been stored in air and the measurements were made in air, so that an oxide layer might be suspected. The penetration distance of X-rays into a surface under total external reflection conditions is known as the evanescent layer thickness. For Si at 10 keV at the critical angle the evanescent layer thickness can be readily computed, albeit for a smooth surface (Tolan, 1999 ▸). The resulting value is 720 Å. We infer that the growth of any possible oxide layer on the smooth sample was considerably less than this evanescent layer thickness and that any oxide layer grew only slowly on the smooth silicon sample.

A further point concerns the diffuse scattering at the specular condition. We found that if longer cut-off values are chosen, that is, if PSD fits are made to reach to shorter values of *q*_*r*_, then a peak in the diffuse scattering at the specular condition can be produced. However, such a peak is broad and not well shaped to match the data (see also the results for the rough sample). Too much intensity adjacent to the specular peak invariably results. The overall data sets could be fit with much improved fidelity by taking shorter cut-offs and invoking a specular term within the BA, as we have done.

**Figure 5:**
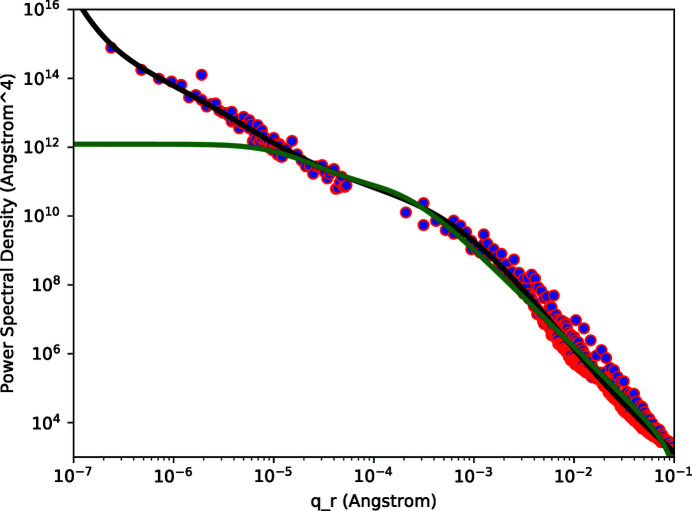
Power spectral density of the rough sample. The data points were obtained from optical interferometry and from atomic force microscopy. The solid black curve is a polynomial fit to the concatenated data set. The solid green curve results from equation (8) applied to the correlation function shown in Fig. 6 ▸.

**Figure 6:**
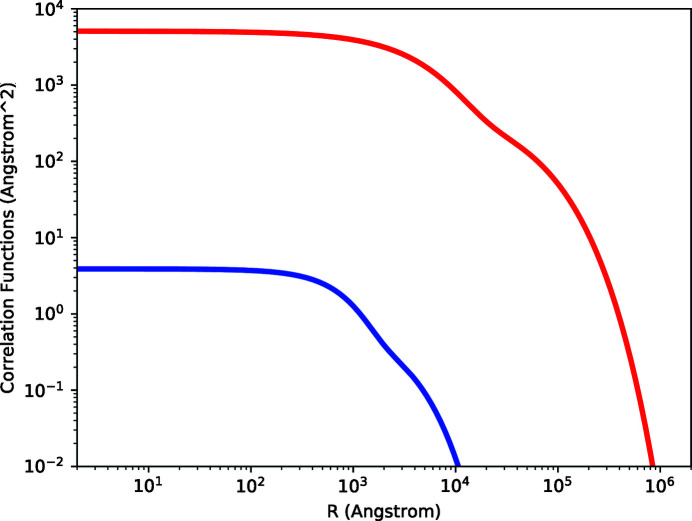
Correlation functions for the smooth (blue) and rough (red) samples obtained by optimizing fits to both: (i) the polynomial fits to the PSD data and (ii) the X-ray diffuse scattering data.

**Figure 7:**
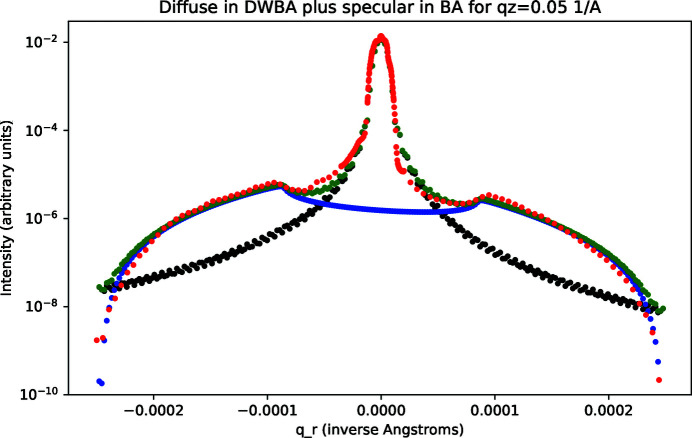
Smooth sample: rocking data and calculated fit (green) for *q*_*z*_ = 0.05 Å^−1^; measured data (red), calculated specular scattering in the BA (black), calculated diffuse in the DWBA (blue), and total calculated scattering (green).

**Figure 9:**
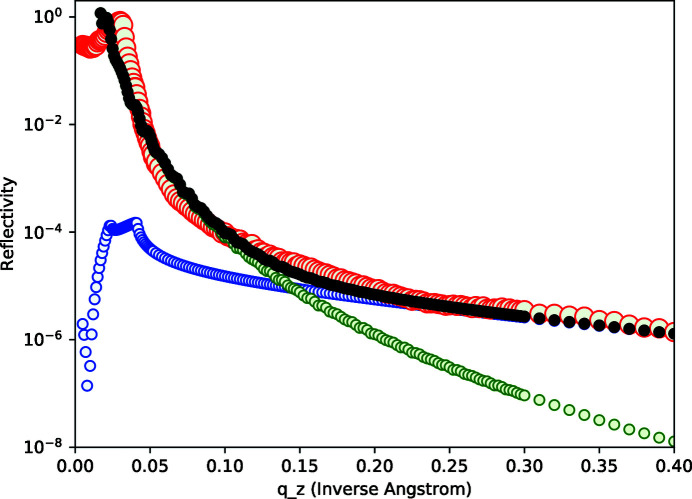
Smooth sample: measured longitudinal scattering data for an angular offset of 0.05° (red), calculated specular scattering in the BA (green), calculated diffuse in the DWBA (blue), and total calculated scattering (black).

**Figure 10:**
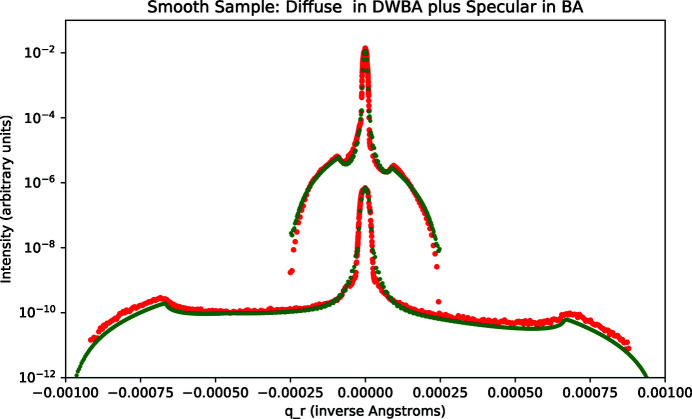
Smooth sample: rocking data (red) and calculated fit (green) for both *q*_*z*_ settings of 0.05 and 0.10 Å^−1^. The offset between the two *q*_*z*_ cases is arbitrary and chosen for clarity.

Of course there is also a specular scattering term within the DWBA. The paper by Sinha *et al.* (1988 ▸) only reports this at the exact specular condition, that is, not away from the exact specular condition as needed to fit our scattering data. We noted that the Nevot-Croce correction to exact specular scattering arising out of the DWBA formalism (Sinha *et al.*, 1988 ▸) at the *q*_*z*_ values we report for the smooth sample is less than 4% [see also Fig. 5 ▸ in the above paper by Sinha *et al.* (1988 ▸) for *q*_*z*_ values of 0.05 and 0.10 Å^−1^]. Consequently, we found it more straightforward to develop fits to our data away from but near the specular peak using the BA. As a check to this procedure, we have also shown that the sum of the BA specular and DWBA diffuse terms agree with the data taken in a longitudinal scan as shown in Fig. 9 ▸.

We consider that the short cut-offs that we invoke are of more general interest since they pertain to the bandpass applicable to diffuse X-ray scattering. Another way to state this conclusion is that X-ray diffuse scattering is not sensitive to roughness that is sometimes referred to as waviness or undulating.

A final point to make is that we can judge the merit of making the so-called ‘low-roughness’ approximation which makes the following approximation in equation (19),

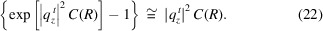

For the smooth sample, we find no change upon making this approximation, which serves to further the conclusion that the smooth sample indeed has very low roughness.

### 8.2. Rough sample

**Figure 11:**
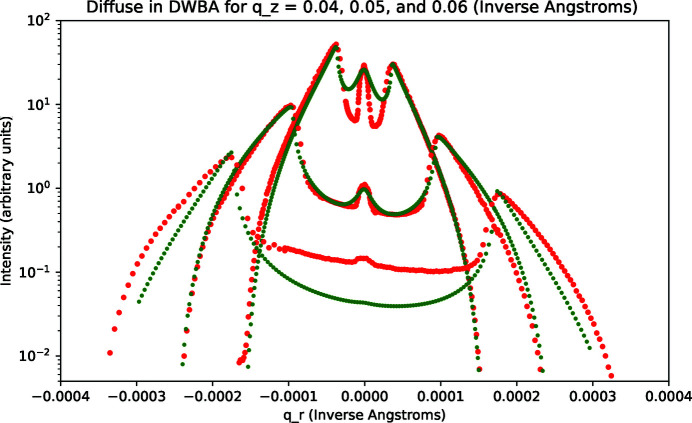
Rough sample: rocking data (red) and calculated fit (green) for *q*_*z*_ = 0.04, 0.05, 0.06 Å^−1^. The offset between the three *q*_*z*_ cases is arbitrary and chosen for clarity. The fit invokes only calculated diffuse scattering in the DWBA.

The measured rocking data for the rough sample were qualitatively different than for the smooth sample. We report results for *q*_*z*_ values of 0.04, 0.05, and 0.06 Å^−1^. The data along with optimal fits are shown in Fig. 11 ▸. The fitting parameters that we applied were obtained by fitting to PSD data as detailed above and are listed in Table 1 ▸. An obvious difference compared with the case of the smooth sample is that the Yoneda wings are much stronger in relation to the intensity at the specular condition. The fits shown in Fig. 11 ▸ are only for diffuse scattering in the DWBA approximation according to equations (15)–(19), that is, a specular term in the BA is not added. Good agreement with the measured data was only obtained at *q*_*z*_ = 0.05 Å^−1^. In the other cases the agreement is poor except near the Yoneda wings.

As for the smooth sample, the position of the Yoneda wings is sensitively dependent on the index of refraction, and we inferred the values in Table 2 ▸. In distinction from the case of the smooth sample, the index of refraction values for the rough sample are consistent with those of amorphous silicon dioxide (Windt, 2007 ▸). We conclude that an oxide layer had developed on the rough sample.

Attempts to add a specular term, in the same manner as was done for the smooth sample, did not result in improved fits. Invariably the *q*_*r*_ region between the Yoneda peaks could not be fit equally well in all three *q*_*z*_ cases. We also attempted fits to the PSD data to smaller values of *q*_*r*_, that is, extending to longer length scales, but these resulted in too much intensity at the specular condition relative to that at the Yoneda wings. Nevertheless, we note that some aspects of the data could be reproduced. Firstly, fits in good agreement with the data were obtained for the Yoneda wings themselves, and, secondly, fits that only invoked diffuse scattering showed very weak peaks at the specular condition, in general agreement with the data.

The DWBA theory of Sinha *et al.* (1988 ▸) was derived to apply for *q*_*z*_σ < 1 and for Gaussian distributed roughness. We determined that the low roughness condition was not satisfied for the rough sample, so one might expect that equations (15)–(19) will not fully suffice. Incorporation of higher-order terms beyond the DWBA is one direction that might improve all the fits. However, also, the roughness may not have been Gaussian distributed in the case of the rough sample. We are not able to shed light on which of these directions might prove more fruitful in improving all the fits.

## 9. Summary

Power spectral density data for the spatial frequencies of surfaces of both a ‘smooth’ and a ‘rough’ silicon sample are reported. These samples were prepared as part of a polishing study for synchrotron X-ray optics. The PSD data were fit by taking the Fourier transform of trial autocorrelation functions. A sum of two self-affine expressions for the trial autocorrelation functions yielded good overall fits to the PSD data. Good fits to the diffuse scattering data at two values of *q*_*z*_ made by applying the DWBA are reported for the smooth sample. The diffuse scattering data were obtained by rocking samples at fixed and low values of *q*_*z*_. Furthermore, good fits in the vicinity of the specular condition were obtained for the smooth sample by incorporating an expression applicable within the BA. Yoneda wings could be well fit in amplitude as well as location for both the smooth and the rough samples. The angular location of the Yoneda peaks were fit to values of the index of refraction which were found to be consistent with a silicon surface in the case of the smooth sample and consistent with a silicon dioxide surface in the case of the rough sample. For the rough sample, however, scattering data in the vicinity of the specular condition could not be equally well fit for all values *q*_*z*_. Unlike for the smooth sample we were not able to improve these fits by adding in a specular scattering term based on the BA. The expressions of Sinha *et al.* (1988 ▸) based on the DWBA that we applied are limited to ‘low roughness’ cases, and we conclude that this condition was satisfied for the smooth sample but not for the rough sample.

## Figures and Tables

**Figure 3:**
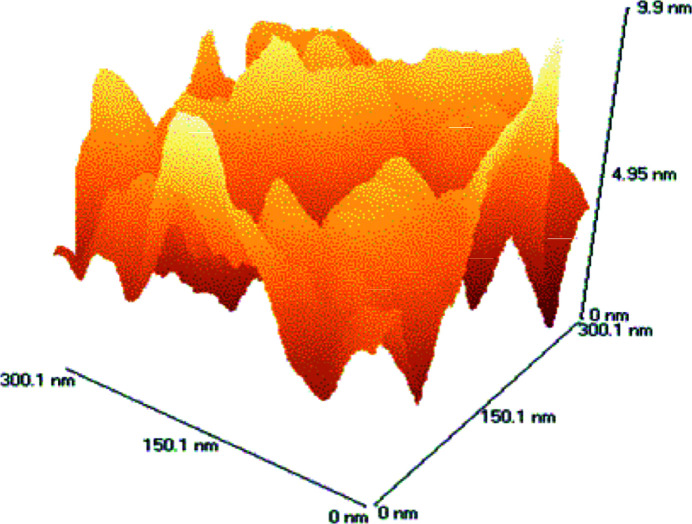
An image of the rough sample obtained with an atomic force microscope.

**Figure 8:**
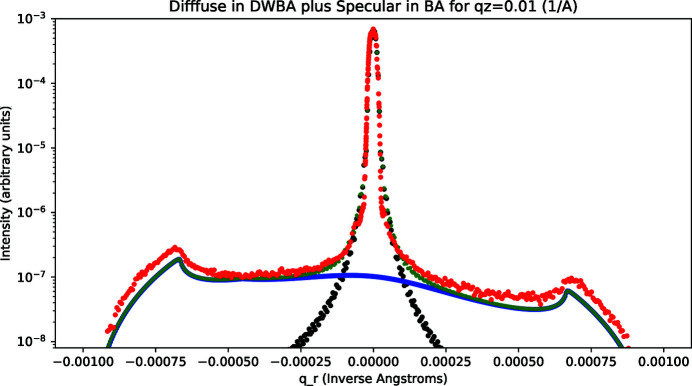
Smooth sample: rocking data and calculated fit (green) for *q*_*z*_ = 0.10 Å^−1^; measured data (red), calculated specular scattering in the BA (black), calculated diffuse in the DWBA (blue), and total calculated scattering (green).

**Table 1:** Fitting parameters to PSD data for the smooth and rough samples

	σ_1_ (Å)	σ_2_ (Å)	ξ_1_ (µm)	ξ_2_ (µm)	*h*_1_	*h*_2_
Smooth	0.95	1.73	0.20	0.08	0.45	0.75
Rough	24.49	67.08	2.75	0.40	0.35	0.45

**Table 2:** Fitting parameters to index of refraction for smooth and rough samples

	δ	β
Smooth	5.2 × 10^−6^	7.7 × 10^−8^
Rough	4.6 × 10^−6^	4.0 × 10^−8^
